# Vagus nerve stimulation for treating refractory epilepsy with myoclonic seizures in children

**DOI:** 10.3389/fneur.2026.1715403

**Published:** 2026-01-27

**Authors:** Guifu Geng, Yao Meng, Wandong Hu, Fang Qi, Jianguo Shi

**Affiliations:** 1 Department of Epilepsy Center, Jinan Children's Hospital, Children's Hospital Affiliated to Shandong University, Jinan, ShanDong, China; 2 Department of Functional Neurosurgery, Jinan Children's Hospital, Children's Hospital Affiliated to Shandong University, Jinan, ShanDong, China

**Keywords:** children, myoclonic seizure, outcome, pediatric epilepsy, vagus nerve stimulation

## Abstract

**Objective:**

To assess the efficacy, tolerability, and safety of vagus nerve stimulation (VNS) in pediatric refractory epilepsy with myoclonic seizures.

**Methods:**

We conducted a retrospective monocentric study at a pediatric center specializing in myoclonic seizures. This study included 19 children (13 males, 6 females; mean age 5.8 years, range: 2–14 years) who underwent VNS implantation between January 2019 and July 2025. Myoclonic seizures were confirmed by video electroencephalogram (v-EEG). The median number of Anti-seizure Medications (ASMs) at implantation was 3.1 (IQR: 2–4). The mean follow-up duration was 31 months (range: 12–56 months).

**Results:**

Patients exhibited various seizure types, including infantile spasms, myoclonic, myoclonic-tonic, generalized tonic–clonic, generalized tonic, and focal seizures. At the last follow-up, 10 patients (52.6%) achieved ≥50% seizure reduction, and 4 (21.1%) attained seizure freedom. The seizure freedom rate was 31.6% for myoclonic seizures.

**Significance:**

VNS demonstrates promise as a safe and effective treatment for pediatric refractory epilepsy (PRE). The seizure freedom rate for myoclonic seizures was particularly noteworthy. These findings suggest that VNS should be considered an early intervention to optimize myoclonic seizure control outcomes.

## Highlights

- Patients commonly have neurodevelopmental delay when epilepsy presents with myoclonic seizures and other seizure types.
- The seizure freedom rate of myoclonic seizures was higher than the rate of all seizure types (31.6% vs. 21.1%) at the last follow-up.
- VNS It may be worth considering this early in the treatment course to maximize benefits for myoclonic seizures control.

## Introduction

Myoclonic seizures are defined as sudden, brief shock-like involuntary movements, typically lasting less than 50 ms in positive myoclonus and up to 500 ms in negative myoclonus, and are usually preceded by epileptiform discharges on electroencephalogram (EEG) (1). In pediatric refractory epilepsy (PRE), myoclonic seizures are observed across various epilepsy syndromes, such as epilepsy with myoclonic atonic seizures (MAE), Lennox–Gastaut syndrome (LGS), and Dravet syndrome (DS) (2, 3). Moreover, a considerable number of patients experience myoclonic seizures as their primary seizure type without fulfilling the diagnostic criteria for a defined syndrome. The prognosis is poor when epilepsy presents with myoclonic seizures and other seizure types (1). Valproate (VPA) is effective in treating myoclonic seizures; however, emerging evidence supports the efficacy of newer ASMs (4). Nevertheless, 35% of cases meet the criteria for intractable myoclonic epilepsy and continue to experience seizures despite the use of multiple antiepileptic medications (5). Treatment options remain limited, as resective surgery is not suitable for generalized myoclonic epilepsy. Although DBS demonstrates greater efficacy than vagus nerve stimulation (VNS) in generalized epilepsy (6), VNS is typically recommended as the initial intervention for children.

VNS is an established adjunctive therapy for children with intractable epilepsy; however, its efficacy in pediatric myoclonic epilepsy requires further investigation (7). VNS warrants consideration as an effective treatment for patients with therapy-resistant generalized epilepsy, with myoclonic epilepsy being the sole exception (8). Previous studies investigating VNS in intractable myoclonic epilepsy have reported limited and inconsistent results (9, 10). Research indicates that VNS for resistant generalized epilepsy achieves only a 28–40% reduction in the frequency of myoclonic seizures (11). VNS has proven beneficial in cases of severe, refractory myoclonic epilepsy, enhancing the quality of life and daily functioning (12). The long-term outcomes of refractory myoclonic seizures treated with VNS remain unclear. This study aims to examine the impact of VNS on PRE with myoclonic seizures, contributing further evidence to its potential role in myoclonic seizure management.

## Patients and methods

### Patient selection

This retrospective study reviewed patients diagnosed with PRE who underwent VNS implantation (G112, PINS Medical, Beijing, China) at the Children’s Hospital of Shandong University between January 2019 and July 2025. The inclusion criteria were as follows: (1) age <18 years at the time of VNS therapy (multiple antiepileptic drugs proved ineffective pre-surgery; patients deemed unsuitable for corpus callosotomy); (2) a minimum follow-up period of 12 months post-VNS therapy; (3) v-EEG confirmation of myoclonic seizures pre-VNS therapy. Patients who had previously undergone epilepsy surgery were excluded.

All participants underwent brain magnetic resonance imaging (MRI) using a 3.0 Tesla SP system (Siemens, Erlangen, Germany) with standardized epilepsy protocols. The imaging protocol included high-resolution T1-weighted volume acquisition, T2-weighted sequences, and fluid attenuated inversion recovery (FLAIR) sequences. MRI findings were categorized as either normal or abnormal.

All children underwent v-EEG monitoring for more than 3 h before VNS therapy. Epileptic seizures were classified according to the ILAE epileptic seizure classification (13), and the EEG background was classified into normal or slow groups. Each patient was evaluated by a multidisciplinary team (MDT) to determine seizure types and guide treatment strategies. The interval between clinical seizure onset and VNS treatment initiation was defined as the time lag, and patients were stratified into short (<3 years) or long (≥3 years) time lag groups.

The clinical data collected included age, gender, epilepsy syndrome, seizure type, age at seizure onset, and seizure frequency. Seizure frequency was categorized as follows: (1) daily seizure, defined as at least one seizure per day; (2) weekly seizure, fewer than one seizure per day but at least one seizure per week; and (3) monthly seizure, fewer than one seizure per week but at least one seizure per month. The collected VNS parameters included the output current (mA) and duty cycle. Data were collected at the final pre-VNS visit and at 6, 12, 24, and 36 months of follow-up.

All patients exhibited developmental delay (DD), which was categorized into three groups: mild DD group (independent movement and communication), moderate DD group (independent movement and partial communication), and severe DD group (completely dependent movement and no communication). Quality of life and seizure severity were assessed based on patient and family reports, as no standardized or validated questionnaires were used.

This study was approved by the ethical committee of the Children’s Hospital Affiliated with Shandong University (SDFE-IRB/P-2024025).

### Programming strategy

Stimulation was initiated 1–2 weeks after VNS implantation. The initial parameters included an output current of 0.2 mA, a signal on time of 30 s, and a signal off time of 5 min. Signal frequency (30 Hz) and pulse width (250 μs) were maintained constant, with the magnet current set 0.3 mA above the output current. Within the first 2 months after discharge, the effective treatment current intensity increased to 1.0–1.5 mA (via clinic or remote programming). Parameters were adjusted by 0.2–0.3 mA increments based on improvements in seizure control and patient tolerance.

### Outcome evaluation

Follow-up and outcome data were collected through a retrospective chart review. The patient’s guardians recorded the seizure frequency on a daily or weekly basis. Additional information on recent seizure activity and current ASM regimens was obtained via telephone interviews with patients, families, or caregivers. For patients who could not be reached, follow-up was censored at the date of the last office visit or inpatient admission. Seizure outcomes were assessed using the VNS-specific classification guideline proposed by McHugh et al. (14). Patients achieving 50% or greater reduction in seizure frequency were classified as responders, and those with less than a 50% reduction in seizure frequency were classified as non-responders.

### Statistical analysis

Data were analyzed using SPSS Statistics 23 (IBM Corp., Armonk, NY, USA). Normally distributed continuous variables are presented as mean and standard deviation. Categorical variables, including sex, seizure frequency, lag time, ASM numbers, and MRI findings, were analyzed using Fisher’s exact test. A *p*-value < 0.05 was considered statistically significant.

## Results

### Patient characteristics

**Figure 1:**
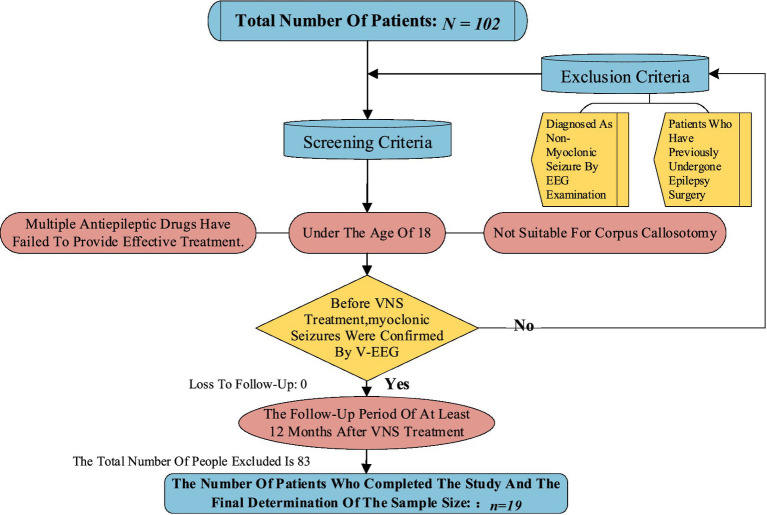
Patient selection process.

The patient selection process is shown in Figure 1. A total of 19 children (13 males, 6 females) with a mean age of 5.8 years (2–14 years) underwent VNS implantation. The mean follow-up period was 31 months (12–56 months). Age at epilepsy onset ranged from 2 to 96 months, with a median of 28.5 months. In addition to myoclonic seizures, V-EEG recordings revealed various seizure types, including infantile spasms, spasm-tonic, myoclonic-tonic, tonic, generalized tonic–clonic, generalized tonic, and focal seizures. Spasm seizures were the most common accompanying symptom (78.9%). Regarding seizure frequency, 14 patients (73.7%) experienced daily seizures. Six children (31.6%) had previously shown no response to the ketogenic diet (KD) before VNS placement (Table 1).

**Table 1:** General characteristics of the study population (*N* = 19).

Characteristic	Value^a^
Sex	
M	13 (68.2%)
F	6 (31.6%)
Age at first seizure, months	28.5 (2–96)
Age at VNS, years	5.8 (2–14)
Duration of epilepsy, years	3.2 (1–9)
Follow-up time, months	31 (12–56)
Other seizure type recorded by v-EEG before VNS	
Spasm	15 (78.9%)
Tonic seizure	8 (42.2%)
Spasm-tonic	6 (31.6%)
Atypical absence seizures	5 (26.3%)
Myoclonic-tonic	4 (21.1%)
Focal seizure	2 (10.5%)
ASMs and treatment were used before VNS	
VPA	17 (89.5%)
LEV	12 (63.2%)
LTG	11 (57.9%)
TMP	9 (47.4%)
Clobazam	7 (36.8%)
PER	5 (26.3%)
CZP	4 (21.1%)
ZNS	3 (15.8%)
VBG	3 (15.8%)
OXC	3 (15.8%)
LCM	2 (10.5%)
KD	6 (31.6%)
ACTH	3 (15.8%)
MRI	
Normal	7 (36.8%)
Encephalomalacia	4 (21.1%)
Ventricular enlargement	4 (21.1%)
Cerebralatrophy	1 (5.2%)
Grey matter heterotopia	1 (5.2%)
Pachygyria	1 (5.2%)
Malformations of cortical development	1 (5.2%)
Frequency of seizures before VNS	
Dayly	14 (73.7%)
Weekly	4 (21.1%)
Monthly	1 (5.2%)
Developmental delay	
Mild	6 (31.6%)
Moderate	6 (31.6%)
Severe	7 (36.8%)
Epilepsy syndrome	
LGS	10 (52.6%)
IESS	4 (21.1%)
Others	5 (26.3%)
Family or neonatal history	
None	13 (68.4%)
Hypoxic–ischemic encephalopathy	2 (10.5%)
Encephalitis	3 (15.8%)
Febrile seizures	1 (5.2%)
EEG background	
Normal	4 (21.1%)
Slow	15 (78.9%)

M, male; F, female; ACTH, adrenocorticotropic hormone; ASM, antiseizure medicine; KD, ketogenic diet; CZP, clonazepam; LCM, lacosamide; LTG, lamotrigine; LEV, levetiracetam; M, male; OXC, oxcarbazepine; PER, perampanel; SZ, seizure; TPM, topiramate; VGB, vigabatrin; VPA, valproic acid; VNS, vagus nerve stimulation; ZNS, zonisamide; LGS, Lennox–Gastaut Syndrome; IESS, infantile epileptic spasms syndrome; EEG, electroencephalogram.

### Preoperative evaluation

Brain MRI evaluation revealed normal results in 7 children (36.7%). Encephalomalacia was present in 4 (21.1%), ventricular enlargement in 4 (21.1%), and pachygyria in 1 (5.2%). Detailed MR findings are presented in Table 1. Scalp EEG monitoring recorded seizures in all patients, with 8 children (42.2%) exhibiting more than 4 seizure types. A normal EEG background was observed in only 4 children (21.1%) (Tables 1, 2).

**Table 2:** Comparison of the initial characteristics of patients’ response to VNS.

Clinical characteristics	Respond, (all seizures) (*n* = 10)	No respond, (all seizures) (*n* = 9)	*p* value	MS reduction >50% (*n* = 9)	MS reduction ≤50% (*n* = 10)	*p* value
Age at surgery						
≤5 years	7	4	0.255	5	6	0.605
>5 years	3	5		4	4	
Age at onset, month						
≤24 month	5	4	0.586	5	4	0.414
>24 month	5	5		4	6	
Sex						
Male	8	5	0.259	7	6	0.370
Female	2	4		2	4	
Lag time						
≤3 years	7	6	0.630	6	7	0.630
>3 years	3	3		3	3	
No. of seizure types						
<4	8	3	0.055	6	5	0.395
≥4	2	6		3	5	
Presence of spasm-tonic						
Yes	4	2	0.370	4	2	0.259
No	6	7		5	8	
No. of ASMs at VNS implant						
≤2	3	2	0.556	3	2	0.556
>2	7	7		7	7	
MRI						
Normal	6	1	0.040#	4	3	0.430
Abnormal	4	8		7	5	
Seizure frequency						
Daily	8	6	0.444	6	8	0.444
<Daily	2	3		3	2	
Follow-up time						
≤24 months	3	5	0.255	2	6	0.115
>24 months	7	4		7	4	
Development delay						
Mild	4	2	0.370	4	2	0.259
Others	6	7		5	8	
VNS parameters						
Output <2.0	7	5	0.430	7	5	0.220
Output ≥2.0	3	4		2	5	
DC < 15%	6	3	0.242	5	4	0.414
DC ≥ 15%	4	6		4	6	

ASM, antiseizure medicine; DC, duty cycle; VNS, vagus nerve stimulation.

### ASM therapy

All patients received three or more major ASMs with limited efficacy. At implantation, the median number of ASMs was 3.1 (IQR: 2–4), and seven children (36.8%) used more than 5 ASMs. The most frequently prescribed ASM at implantation was VPA (89.5%, *n* = 17), followed by topiramate (TPM) (42.1%, *n* = 8) and lamotrigine (LTG) (36.8%, *n* = 7). At the final follow-up, ASM numbers decreased in 8 children (42.1%), 1 patient (5.2%) discontinued all ASMs, 16 children (84.2%) maintained VPA, 7 children (36.8%) added clobazam with one achieving >95% seizure reduction (patient 3), and 5 children (26.3%) modified ASMs with one achieving six-month seizure freedom (patient 10). The final median ASM number was 2.2 (IQR: 0–4).

### VNS outcomes

At the final follow-up, 10 children (52.6%) achieved a ≥ 50% reduction in seizure, and 4 children (21.1%) achieved complete seizure freedom. The McHugh and modified Engel seizure outcome classifications were used to assess the final follow-up outcomes (Table 3). According to the modified Engel scale, 4 children (21.1%) were classified as class I, 1 child (5.2%) as class II, 5 children (26.3%) as class III, and 9 children (47.4%) as class IV. The McHugh scale indicated that 5 children (26.3%) were classified as class I, 5 children (26.3%) as class II, 8 children (42.2%) as class III, and 1 child (5.2%) as class IV–V (Table 3). The rate of seizure freedom for myoclonic seizures exceeded that of all seizure types (31.6% vs. 21.1%) (Table 3). No clinical factors were significantly associated with the VNS response in myoclonic seizures.

**Table 3:** Seizure outcome evaluated by modified Engel and McHugh classification at last follow-up (>1 year).

Class	Engel description	No. of patients (%) (myoclonic seizure)	95%CI	No. of patients (%) (all seizure types)	95%CI
I	Seizure-free; rare, nondisabling SPS	6 (31.6%)	12.58–56.55%	4 (21.1%)	6.05–45.57%
II	>90% reduction in seizure frequency; rare CPS	0	16.29–61.64%	1 (5.2%)	12.58–56.55%
III	50–90% reduction in seizure frequency	7 (36.8%)		5 (26.3%)	
IV	<50% reduction in seizure frequency	6 (31.6%)	12.58–56.55%	9 (47.4%)	24.48–71.14%
V	/				

McHugh description	No. of patients (%) (myoclonic seizure)	95%CI	No. of patients (%) (all seizure types)	95%CI
80–100% reduction in seizure frequency	7 (36.8%)	16.29–61.64%	5 (26.3%)	9.15–51.20%
50–79% reduction in seizure frequency	6 (31.6%)	12.58–56.55%	5 (26.3%)	
<50% reduction in seizure frequency	5 (26.3%)	12.58–56.55%	8 (42.2%)	24.48–71.14%
Magnet benefits only	0		0	
No improvement	1 (5.2%)		1 (5.2%)	

SPS, simple partial seizure; CPS, complex partial seizure.

Sex and age at seizure onset were not significant predictors of VNS treatment response for all seizure types in the univariable analysis. No significant differences were observed in age at surgery, lag time, ASMs used, seizure frequency, or follow-up time. Regarding VNS parameters, treatment outcomes did not differ between patients and high versus low pulse amplitude (output) or duty cycle. The only factor significantly associated with a favorable VNS response was a normal brain MRI (*p* < 0.05). Multiple seizure types may be significantly associated with poor response (*p* = 0.055) (Table 2).

No significant adverse effects were observed in any patient. Only 1 child (5.2%) experienced VNS-related lead pain.

### Neuropsychological outcome

The quality of life assessment was conducted through interviews with patients and their families. All children exhibited DD, with 6 (31.6%) classified as mild, 6 (31.6%) as moderate, and 7 (36.8%) as severe. Comparing baseline status to assessments at 12 months post-VNS therapy, 3 children (15.8%) showed no significant improvement in behavioral and cognitive abilities, and 1 child (5.2%) demonstrated regression in movement (patient 2) (Table 4).

**Table 4:** ASM and VNS parameters at the last follow-up.

Pt.	Sex	Duration of SZ, years	ASMs at VNS implant	ASMs at last FU	VNS parameters at last FU	Myoclonic seizure reduction %	Respond, (all seizure type reduction %)
1	M	3	TPM, LTG, clobazam	LTG	Output, 1.2 mA; Pulse width, 250 μs; SF, 30 Hz; DC, 10%	100%	Yes, SZ free>12 months
2	M	2	TPM, VPA	LTG, VPA,	Output, 1.6 mA; Pulse width, 250 μs; SF, 30 Hz; DC, 10%	100%	Yes, >50%
3	M	5	LEV, VPA, CZP	VPA, PER, clobazam	Output, 1.7 mA; Pulse width, 250 μs; SF, 30 Hz; DC, 17%	100%	Yes, >95%
4	F	3	VPA, CZP	VPA, clobazam	Output, 1.8 mA; Pulse width, 750 μs; SF, 30 Hz; DC, 10%	50%	Yes, >70%
5	F	1	VPA, LEV, TPM	VPA, LEV, clobazam	Output, 1.7 mA; Pulse width, 750 μs; SF, 30 Hz; DC, 17%	60%	No <50%
6	M	4	TPM, LEV, VPA, PER	VPA, PER, LTG, clobazam	Output, 1.8 mA; Pulse width, 500 μs; SF, 30 Hz; DC, 10%	20%	No <30%
7	F	2	TPM, LTG, LCM, CZP	Clobazam, VGB	Output, 2.2 mA; Pulse width, 250 μs; SF, 30 Hz; DC, 17%	80%	Yes >60%
8	M	2	VPA, TPM, LEV	0	Output, 1.2 mA; Pulse width, 250 μs; SF, 30 Hz; DC, 10%	100%	Yes, SZ free>12 months
9	F	3	VPA, TPM	VPA, LEV, clobazam	Output, 0.5 mA; Pulse width, 500 μs; SF, 30 Hz; DC, 17%	50%	No <50%
10	M	3	OXC, VPA, PER	LEV, VPA, clobazam	Output, 0.5 mA; Pulse width, 500 μs; SF, 30 Hz; DC, 17%	100%	Yes, SZ free>6 months
11	F	3	VPA, LEV, ZNS, clobazam	VPA, LEV	Output, 2.3 mA; Pulse width, 500 μs; SF, 30 Hz; DC, 17%	30%	No <50%
12	M	5	VPA, TPM, clobazam	VPA	Output, 0.2 mA; Pulse width, 250 μs; SF, 30 Hz; DC, 10%	100%	Yes, SZ free>6 months
13	M	4	VPA, LEV, LTG, clobazam	LTG, clobazam	Output, 2.0 mA; Pulse width, 500 μs; SF, 30 Hz; DC, 17%	50%	Yes >70%
14	M	5	VPA, LTG, LEV, ZNS	VPA, LTG, CZP	Output, 2.5 mA; Pulse width, 250 μs; SF, 30 Hz; DC, 10%	60%	No <50%
15	M	1	VPA, LTG, clobazam	VPA, LTG, clobazam	Output,0.7 mA; Pulse width, 250 μs; SF, 30 Hz; DC, 28%	50%	No <50%
16	M	3	VPA, clobazam	VPA, clobazam	Output, 2.5 mA; Pulse width, 500 μs; SF, 30 Hz; DC, 28%	40%	No <50%
17	M	9	VPA, LEV, LTG	VPA, LEV, LTG	Output, 2.0 mA; Pulse width, 250 μs; SF, 30 Hz; DC, 17%	0	No improvement
18	M	1	VPA, clobazam, PER	VPA, LTG	Output, 2.0 mA; Pulse width, 250 μs; SF, 30 Hz; DC, 10%	40%	Yes >50%
19	F	2	VPA, LTG, ZNS	VPA, LTG	Output, 1.0 mA; Pulse width, 250 μs; SF, 30 Hz; DC, 10%	30%	No <30%

Pt, patient; ASM, antiseizure medicine; DC, duty cycle; SF, Signal frequency; F, female; FU, follow-up; HIE, hypoxic–ischemic encephalopathy; KD, ketogenic diet; CZP, clonazepam; LCM, lacosamide; LTG, lamotrigine; LEV, levetiracetam; M, male; OXC, oxcarbazepine; PER, perampanel; SZ, seizure; TPM, topiramate; VGB, vigabatrin; VPA, valproic acid; VNS, vagus nerve stimulation; ZNS, zonisamide.

## Discussion

VNS is an established treatment for drug-resistant epilepsy in children (8). Our findings indicate that adjunctive VNS therapy was effective in the treatment of PRE with myoclonic seizures. Overall, 21.1% of children achieved seizure freedom, and 52.6% experienced a 50% or more reduction in seizure frequency. The rate of seizure freedom for myoclonic seizures was 31.6%. When comparing seizure types, VNS appeared to be more effective in reducing generalized tonic–clonic seizures than myoclonic seizures (9). Previous studies on VNS in intractable myoclonic epilepsy have reported modest and inconsistent results (9, 10, 15), with only a 28–40% reduction in myoclonic seizures, lower than other seizure types.

Seizure freedom is widely regarded as the primary predictor of quality of life in patients with epilepsy. Complete seizure freedom is rarely achieved (<10%) in patients with epilepsy receiving VNS therapy (8, 16, 17). In our study of 19 patients with myoclonic seizures, 4 (21.1%) achieved seizure freedom at the final follow-up, exceeding the rates reported in the general epilepsy population.

Developmental and epileptic encephalopathies (DEEs) with myoclonic seizures are often associated with poor cognitive outcomes and persistent refractory seizures. VNS demonstrated good tolerability in patients with DEEs, leading to reduced seizure frequency across all types, particularly those causing falls or drops (18). VNS proved effective for all seizure types in LGS patients. Our study revealed LGS as the most common epilepsy syndrome (*n* = 10), with 2 patients (20%) achieving seizure freedom and 5 (50%) responding to VNS, consistent with the findings of the current research (18).

Long-term neurostimulation follow-up typically shows progressively increasing benefits over time. Previous studies indicate that the duration of stimulation is a significant factor in long-term clinical improvement, due to the cumulative effects of continuous electrical vagus nerve stimulation (19). In our series, although some patients exhibited a positive response after device implantation, outcomes did not show further improvement over time, with no significant differences between patients followed for >24 months and those with ≤24 months of follow-up (Table 2). While younger patients typically demonstrate better cognitive and quality of life outcomes (20), and shorter pre-implant epilepsy durations correlate with improved VNS response rates (21, 22), our study found no significant age difference between responders and non-responders at the final follow-up.

The identification of factors predictive of VNS response is crucial for patient selection, treatment stratification, and stimulation parameter optimization. However, reliable predictors of VNS response remain limited in clinical practice (23). Research has demonstrated that VNS stimulation activates the thalamus, with increased activation correlating with improved seizure control (24). Recent studies have highlighted the central role of the thalamus in myoclonic seizures (25). Enhanced effective connectivity from the total cortex to the thalamus has been observed in patients with myoclonic epilepsy compared with healthy controls (26). In our study, the complete reduction rate of myoclonic seizures exceeded the rate across all seizure types (31.6% vs. 21.2%). Multimodal analyses have indicated that genetically determined dysfunctions of visuomotor coordination and linguistic communication are key mechanisms underlying generalized myoclonic epilepsy (27). Studies have demonstrated enhanced thalamocortical connectivity and progressive thalamic atrophy in myoclonic epilepsy (28). VNS-induced thalamic activation has been associated with seizure response (29). This mechanism may explain the favorable outcomes for myoclonic seizures observed in this study.

A comprehensive analysis has demonstrated that responder rates are significantly higher in patients receiving high-level stimulation than in those with low-level stimulation (30). The high-pulse-amplitude mode appears to be more effective for focal seizures, whereas a high duty cycle mode may prove more effective for epileptic spasms (31). Our study revealed no significant differences in VNS parameters between responders and non-responders at the final follow-up.

The relationship between MRI results and VNS outcome measures has been inconsistent. Some studies have reported that after 6 months of VNS, no significant outcome differences existed between patients with abnormal and normal MRI; however, after 12 months, abnormal MRI groups demonstrated significantly higher VNS responses compared to normal-MRI patients (32, 33). Conversely, other studies found no significant association between the presence of MRI lesions and VNS outcome (34). In our study, patients with normal MRI exhibited better treatment responses, consistent with a recent study (35) that found more responders in the normal MRI group (62%) compared to the lesional group (40%).

The adverse effects of VNS, primarily wound infection and hoarseness, are typically related to the surgical procedure and occur in approximately 1% of patients. No significant adverse effects were observed in our cohort. Only 1 child (5.2%) developed pain related to the VNS lead. Hoarseness and cough may present detection challenges in children with severe developmental delay.

### Limitations and future directions

Our study has several limitations: (1) The inclusion criteria’s focus on v-EEG recorded before VNS prevents identification of all patients with a myoclonic seizure history without an EEG. Clinical assessments of emotional state, neurological deficits, and quality of life were not conducted, despite their known importance in evaluating PRE treatment efficacy. (2) Data regarding pre- and post-implant frequency of emergency room visits and hospitalizations were unavailable for analysis, though previous studies have demonstrated improvements in these measures. (3) The retrospective nature of the study and relatively small sample size introduce inherent biases, suggesting the need for larger prospective studies to establish more definitive conclusions.

## Conclusion

Vagus nerve stimulator therapy is a viable treatment option for PRE. We found that 21.1% of children achieved complete seizure freedom, and 52.6% experienced at least a 50% reduction in seizure frequency. The seizure freedom rate for myoclonic seizures reached 47.4%. These findings support the early integration of VNS into treatment strategies to maximize the benefits of myoclonic seizures control. Future prospective studies should focus on collecting seizure frequency data for each seizure type or obtaining pre- and post-vagus nerve stimulator prolonged video EEG to quantify improvements in myoclonic seizures and epileptiform activity.

## Data Availability

The original contributions presented in the study are included in the article/supplementary material, further inquiries can be directed to the corresponding author.
